# Hypoxia Treatment of Adipose Mesenchymal Stem Cells Promotes the Growth of Dermal Papilla Cells via *HIF-1α* and ERK1/2 Signaling Pathways

**DOI:** 10.3390/ijms241311198

**Published:** 2023-07-07

**Authors:** Qing Wang, Mei Zhou, Hongyan Zhang, Zhuang Hou, Dongjun Liu

**Affiliations:** State Key Laboratory of Reproductive Regulation & Breeding of Grassland Livestock, Inner Mongolia University, Hohhot 010021, China; nmwangqing1991@sina.com (Q.W.); 18586026786@163.com (M.Z.); 18004853744@163.com (H.Z.); 15326028115@163.com (Z.H.)

**Keywords:** hypoxia, hair follicles, adipose mesenchymal stem cells, dermal papilla cells, growth factor, *HIF-1α*

## Abstract

Dermal papilla cells (DPCs) cultured in vitro induce hair follicle formation. Using a hypoxic microenvironment to culture adipose mesenchymal stem cells (ADSCs) can promote hair follicle growth. However, the exact molecular mechanisms underlying this process remain unclear. In this study, ADSCs and DPCs from Arbas Cashmere goats were used. A hypoxic microenvironment promoted the proliferation of ADSCs and increased the pluripotency of ADSCs. The growth factors vascular endothelial growth factor (VEGF), basic fibroblast growth factor (bFGF), and platelet-derived growth factor (PDGF) were upregulated in ADSCs in the hypoxia-conditioned medium (Hypo-cm). Hypo-cm also enhanced the ability of DPCs to induce hair follicle formation. Inhibitors of the ERK1/2 signaling pathway caused the expressions of growth factors that increased in hypoxic microenvironments to decrease; moreover, hypoxia-inducible factor-1α (*HIF-1α*) increased the expression levels of VEGF, bFGF, and PDGF and inhibited the expression of bone morphogenic protein 7 (*BMP7*). In conclusion, these findings improve the theoretical basis for the development of gene therapy drugs for the treatment of alopecia areata and hair thinning.

## 1. Introduction

Dermal papilla cells (DPCs) are highly specialized fibroblasts derived from dermal mesenchyme, a globular structure at the base of the hair follicle that induces follicular growth [1]. During transplantation, the efficiency of follicle formation correlates with the ability of DPCs to induce follicular regeneration [2]. DPCs strongly influence the cyclic alteration of hair follicles [3,4], and even in vitro DPC cultures retain an ability to induce follicle formation and promote hair follicle growth [5]. Hair follicle growth is also closely related to adipose mesenchymal stem cells (ADSCs), which surround the outer layer of DPCs to optimize the follicular microenvironment for hair follicle growth [6,7]. A study transplanted ADSCs with *CD34* markers into mouse fetal epidermal and dermal cells during hair follicle growth, resulting in the detection of ADSCs in the formation of hair follicles, blood vessels, and adipose tissue, which suggested that ADSCs influence hair follicle morphogenesis [8]. In addition, the subcutaneous injection of the adipose-mesenchymal-stem-cell-conditioned medium (ADSC-CM) into the shaved skin of mice showed skin darkening; furthermore, the effect was more pronounced under hypoxic conditions [9]. ADSCs secrete several growth factors that are involved in and regulate follicle growth, and their expression is enhanced in a hypoxic microenvironment [10,11,12]. An ADSC-CM added to a medium of DPCs or endo- and exo-root sheath cells can enhance the viability of these cells, and the expression of several growth factors can be detected in ADSC culture supernatants [9,13,14].

Overall, the regulatory mechanisms of ADSCs on DPCs in hypoxic microenvironments require further investigation. Because the paracrine function of ADSCs strongly influences hair follicle growth, we hypothesized that ADSCs in the hypoxic microenvironment may regulate the proliferation of DPCs and the ability to induce hair follicle regeneration via a paracrine function. To test our hypothesis, we established a hypoxic culture model of ADSCs in which ADSCs were cultured with physical hypoxia and hypoxia mimics. Among the hypoxic mimics, desferrioxamine (DFO) and cobalt chloride (CoCl_2_) were selected because they mimic the hypoxic environment in vivo by inhibiting the activity of proline hydroxylase mainly by binding to Fe^2+^ and thus reducing the degradation of *HIF-1α* under normoxia [15,16]. *HIF-1α*, the main transcription factor in the hypoxia pathway, is normally degraded by the recognition of the ubiquitin ligase complex owing to the hydroxylation of proline hydroxylase; however, under hypoxic conditions, the generated free radicals can inhibit the catalytic activity of proline hydroxylase [17], resulting in an increased *HIF-1α* expression.

Alkaline phosphatase (*ALP*) is an important indicator of follicle formation induced by DPCs, and the ability of DPCs to induce follicle formation correlates with altered *ALP* activity [4,18,19]. *SOX2* determines the type of follicle growth and participates in skin reconstruction, playing an important role in follicle growth [20]. Alpha-smooth muscle actin *(α-SMA*) is associated with myofibroblast transformation originating from hair follicle cells [21,22]. Neural cell adhesion molecule (*NCAM*) can mediate intercellular and intercellular matrix adhesion [23], which is presumed to be related to the aggregation of DPCs in vivo. The *versican* plays an important role in maintaining and promoting the growth of DPCs and the initiation of the hair follicle cycle [24]. We determined whether the ability of DPCs to induce hair follicle formation was altered by examining the expression of specific proteins associated with hair follicle formation in DPCs. VEGF, PDGF, and bFGF can promote hair follicle growth during hair follicle growth, among which VEGF controls hair growth and follicle size by promoting angiogenesis [25]. PDGF and its receptors regulate hair follicle stem cell activity and induce the onset of initial hair follicle growth in mice [26]. FGF2 and PDGF have synergistic effects in promoting the proliferation and maintenance of the inductive capacity of DPCs [27]. Our study demonstrated that the expressions of growth factors VEGF, PDGF, and FGF2 were up-regulated in the conditioned medium of ADSCs. BMPs belong to the TGF-β superfamily and strongly influence the development of other tissues and organs as well as promote bone formation [28]. Also, *BMP7* affects the differentiation of the hair follicle trunk after birth [29,30]. Our study demonstrated that the expressions of growth factors VEGF, PDGF, and FGF2 were up-regulated in the conditioned medium of ADSCs. Also, the MAPK/ERK signaling pathway and *HIF-1α* co-regulated the regulation of DPCs via ADSCs in a hypoxic microenvironment. This provides a new reference for other potential ways in which ADSCs affect hair follicle growth in a hypoxic microenvironment.

## 2. Results

### 2.1. Establishment of a Hypoxic Microenvironment

**Figure 1:**
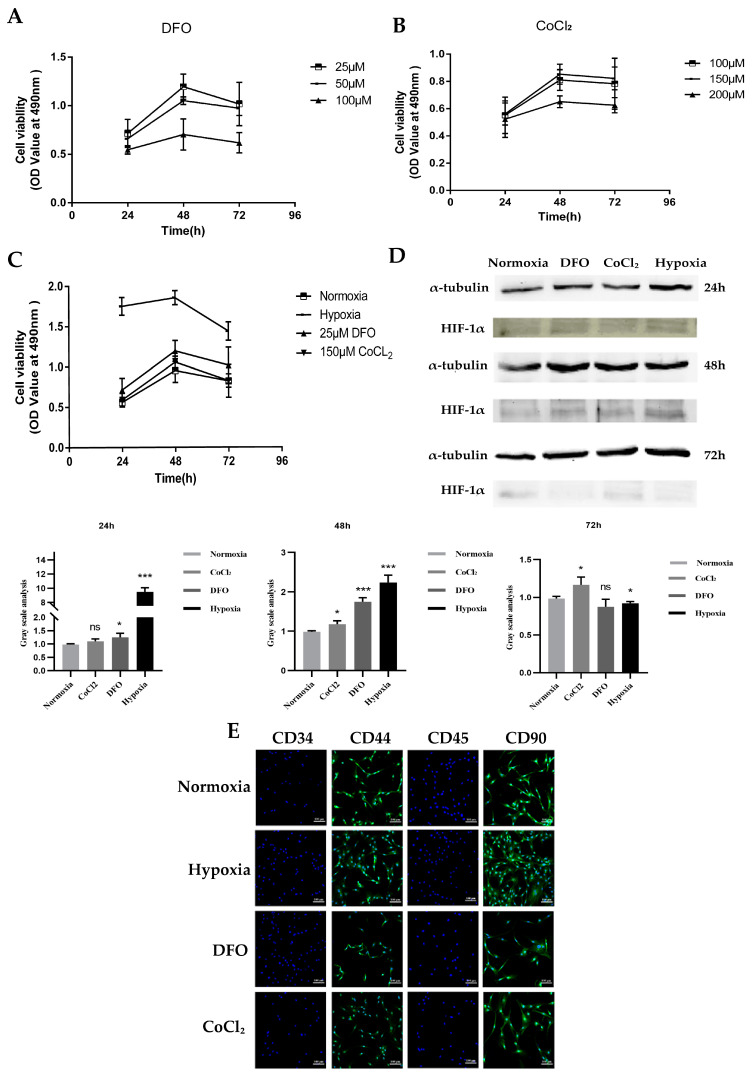
Establishment of a hypoxic microenvironment. (**A**) Effects of DFO at different concentrations on activity of ADSCs after 24, 48, and 72 h cultivation. N = 3. (**B**) Effects of different concentrations of CoCl_2_ on the activity of ADSCs after 24, 48, and 72 h cultivation. N = 3. (**C**) Activity of ADSCs cultured under 25 μM DFO, 150 μM CoCl_2_, normoxia, and physical hypoxia for 48 h. N = 3. (**D**) *HIF-1α* gene expression in ADSCs after 24, 48, and 72 h of treatment with different hypoxia methods. N = 3. (**E**) The expressions of cell surface markers *CD34*, *CD44*, *CD45*, and *CD90* (scale: 100 μm) were detected using immunofluorescence after culturing ADSCs with 25 μM DFO, 150 μM CoCl_2_, and physical hypoxia for 48 h. * *p* > 0.05; *** *p* < 0.01; ns, not significant compared with ADSCs cultured under normal conditions. ADSCs, adipose mesenchymal stem cells; DFO, desferrioxamine.

We treated the cells with physical hypoxia and hypoxic mimetics DFO and CoCl_2_. The proliferative activity of ADSCs was detected with a Cell Counting Kit-8 (CCK-8) after continuous treatment of ADSCs for 24, 48, and 72 h using different concentrations of DFO (25, 50, and 100 μM) and CoCl_2_ (100, 150, and 200 μM). Among them, ADSCs treated with 25 μM DFO and 150 μM CoCl_2_ for 48 h showed the highest cell activity (Figure 1A,B). The concentration and time were selected as the optimal treatment conditions for subsequent experiments. The effects of the four culture methods on ADSC proliferation were examined. The physically hypoxic culture had the highest cell activity, and the cell activity in the 25 μM DFO group was greater than that in the 150 μM CoCl_2_ group. However, cell activity was higher in both the physical hypoxic culture and hypoxic mimetic culture than in the normoxic culture (Figure 1C). To characterize the established hypoxic model of ADSCs, the protein expression level of the hypoxia-inducible factor *HIF-1α* was detected using Western blot. Compared with the control group of ADSCs cultured in untreated normoxia, *HIF-1α* expression following 24 h of hypoxia treatment was higher in both the physical hypoxia and drug mimic groups (25 μM DFO and 150 μM CoCl_2_, respectively). Over time, the *HIF-1α* expression became the highest after 48 h of hypoxia and decreased after 72 h (Figure 1D). This indicated that the model of physical hypoxia and the drug mimic group (25 μM DFO and 150 μM CoCl_2_) treatment for 48 h was established. The surface markers of ADSCs cultured for 48 h in physical hypoxia and drug mimetic groups (25 μM DFO and 150 μM CoCl_2_) were identified using cellular immunofluorescence and consistent with the normoxic culture, the expressions of ADSCs marker genes CD44 and CD90 were detected, and hematopoietic stem cell marker genes CD34 and CD45 were not detected (Figure 1E). The hypoxia model established via either physical hypoxia or hypoxic mimics did not change the basic characteristics of ADSCs.

### 2.2. Hypoxic Microenvironment Enhances the Proliferation and Paracrine Level of ADSCs

**Figure 2:**
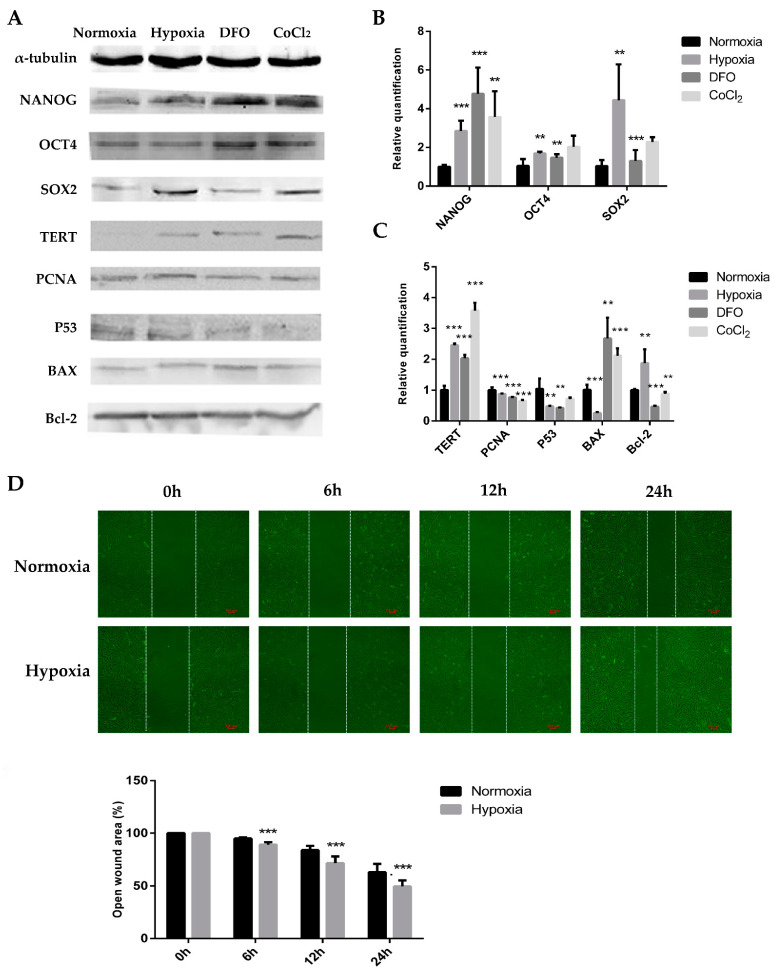
Hypoxic microenvironment enhances the proliferation and paracrine level of ADSCs. (**A**) Western blot was used to detect the expressions of *NANOG*, *OCT4*, *SOX2*, *TERT*, *PCNA*, *p53*, *BAX*, and *Bcl-2* in ADSCs treated with 25 μM DFO, 150 μM CoCl_2_, or after physical hypoxia for 48 h. N = 3. (**B**) qRT-PCR was used to detect the expressions of *NANOG*, *OCT4*, and *SOX2* in ADSCs treated with 25 μM DFO, 150 μM CoCl_2_, or after physical hypoxia for 48 h. N = 3. (**C**) Expressions of proliferation-related genes *TERT* and *PCNA* and apoptosis-related genes *p53*, *BAX*, and *Bcl-2*, detected with qRT-PCR in ADSCs treated with 25 μM DFO, 150 μM CoCl_2_, or after physical hypoxia for 48 h. N = 3. (**D**) Cell migration after 6, 12, and 24 h of culture in hypoxic and normoxic environments. Image J software was used to analyze the ratio of scratch area to baseline area after 6, 12, and 24 h culture in hypoxic and normoxic environments. N = 3. ** 0.01 < *p* < 0.05; *** *p* < 0.01 compared with ADSCs cultured under normal conditions. ADSCs, adipose mesenchymal stem cells; DFO, desferrioxamine; Hypo-cm, physically hypoxic culture; Nor-cm, normoxic cultured; qRT-PCR, quantitative reverse-transcription PCR.

To investigate the effect of hypoxic culture on the function of adipose MSCs, we studied the expression of pluripotency genes. We observed that the expressions of *NANOG*, *OCT4*, and *SOX2* were significantly higher following hypoxic culture conditions compared to those of ADSCs cultured in normoxia (Figure 2A,B). *NANOG* and *OCT4* levels were elevated more significantly in the DFO and CoCl_2_ groups, and *SOX2* levels were elevated more significantly in the physical hypoxia and CoCl_2_ groups. This indicates that hypoxic culture improved the pluripotency of stem ADSCs. The transcript and protein expressions of ADSC proliferation and apoptosis-related genes were also examined following hypoxic culturing in different ways. The telomerase reverse transcriptase (*TERT*) expression increased in the hypoxia treatment groups, whereas proliferating cell nuclear antigen (*PCNA*) expression increased in the physical hypoxia group and decreased in the DFO and CoCl_2_ groups. *P53* expression decreased in the hypoxia treatment groups, *BAX* expression decreased in the physical hypoxia group and increased in the DFO and CoCl_2_ groups, and *Bcl-2* expression increased in the physical hypoxia group and decreased in the DFO and CoCl_2_ groups (Figure 2A,C). The *Bcl-2*/*BAX* ratio increased in the physical hypoxia group, both at the transcriptional and protein levels. The decrease in *p53* expression and increase in the *Bcl-2*/*BAX* ratio further demonstrated the promotion of the hypoxic microenvironment on ADSC proliferation. Since the physical hypoxic approach had a greater effect on anti-apoptosis, subsequent studies on the effect of the hypoxic microenvironment on the cell function of ADSCs were performed under the physical hypoxic approach.

**Figure 3:**
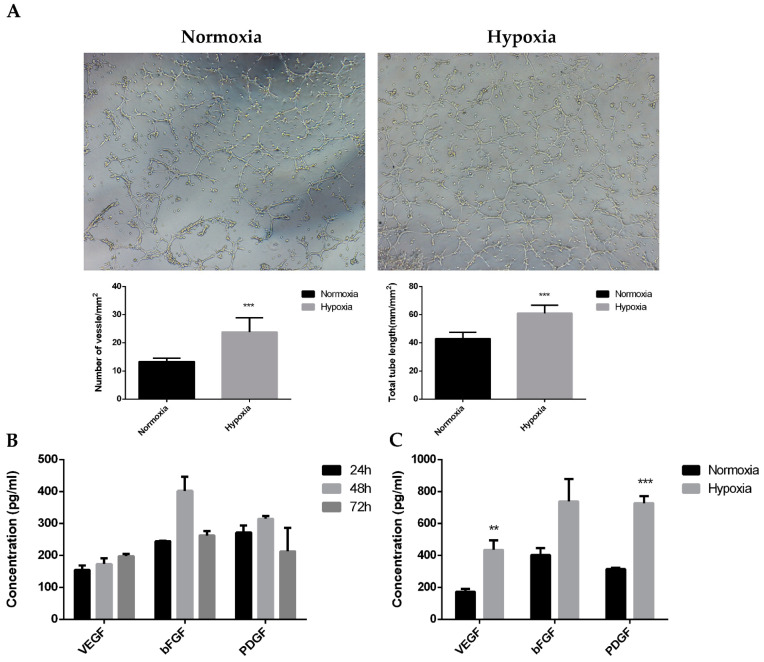
Hypoxic microenvironment enhances the proliferation and paracrine level of ADSCs. (**A**) Cavity-like structure of ADSCs formed in hypoxic and normoxic environment. Image J software was used to analyze the number and length of blood vessels formed per unit area of ADSCs in hypoxic and normoxic environments. N = 3. (**B**) The expressions of VEGF, bFGF, and PDGF in Norcm after 24, 48, and 72 h. N = 3. (**C**) Changes in the expression levels of VEGF, bFGF, and PDGF in Nor-cm and Hypo-cm after 48 h. N = 3. ** 0.01 < *p* < 0.05; *** *p* < 0.01 compared with ADSCs cultured under normal conditions.

The area between the cell scratches gradually decreased after 6, 12, and 24 h. The ratio of the scratch area to the starting area of ADSCs was always smaller after hypoxic culture than after normoxic culture, and the difference was significant after 24 h (Figure 2D). This confirmed that the migration ability of the cells was stronger after hypoxic culturing. Thus, the hypoxic microenvironment stimulated ADSC migration. The angiogenesis assay results revealed that the number and length of blood vessels per unit area were significantly higher in ADSCs after hypoxic culture than after untreated normoxic culture (Figure 3A). Conditioned media were collected from normoxic (Nor-cm) cultured ADSCs for 24, 48, and 72 h, and the expression of growth factors was detected via ELISA. The expressions of growth factors *VEGF*, *bFGF*, and *PDGF* were higher in the conditioned medium at 48 h; therefore, 48 h was used as the final collection time (Figure 3B). Conditioned media from the physically hypoxic cultured (Hypo-cm) ADSC at 48 h was collected, and the concentration of growth factors was examined. The concentrations of *VEGF*, *bFGF*, and *PDGF* were higher in Hypo-cm than in Nor-cm ADSCs (Figure 3C).

### 2.3. Conditioned Medium for ADSCs Enhances Proliferation and Induces Follicle Formation in DPCs

**Figure 4:**
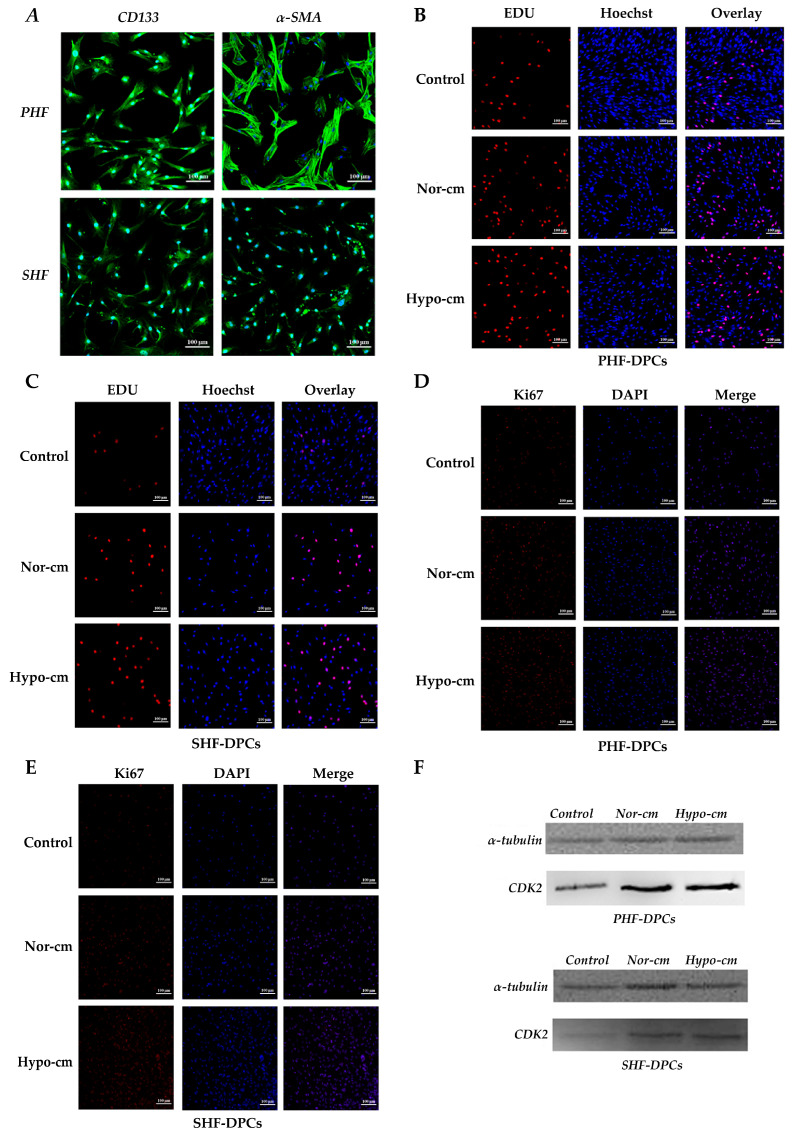
Conditioned medium for ADSCs enhances proliferation and induces follicle formation in DPCs. (**A**) Immunofluorescence was used to detect the expression of *CD133* and α-SMA on the surface markers of PHF-DPCs and SHF-DPCs (scale: 100 μm). (**B**,**C**) EdU was used to detect the change in proliferation ability of PHF-DPCs and SHF-DPCs after using conditioned medium Hypo-cm and Nor-cm. (**D**,**E**) Immunofluorescence was used to detect the expression of Ki67 after using conditioned medium Hypo-cm and Nor-cm for PHF-DPCs and SHF-DPCs (scale: 100 μm). (**F**) Results of Western blot to detect the expression of CDK2 after culturing PHF-DPCs and SHF-DPCs in Hypo-cm and Nor-cm for 48 h. N = 3. Hypo-cm, physically hypoxic culture; Nor-cm, normoxic cultured; PHF-DPCs, primary hair follicle–dermal papilla cells; SHF-DPCs, secondary hair follicle–dermal papilla cells.

**Figure 5:**
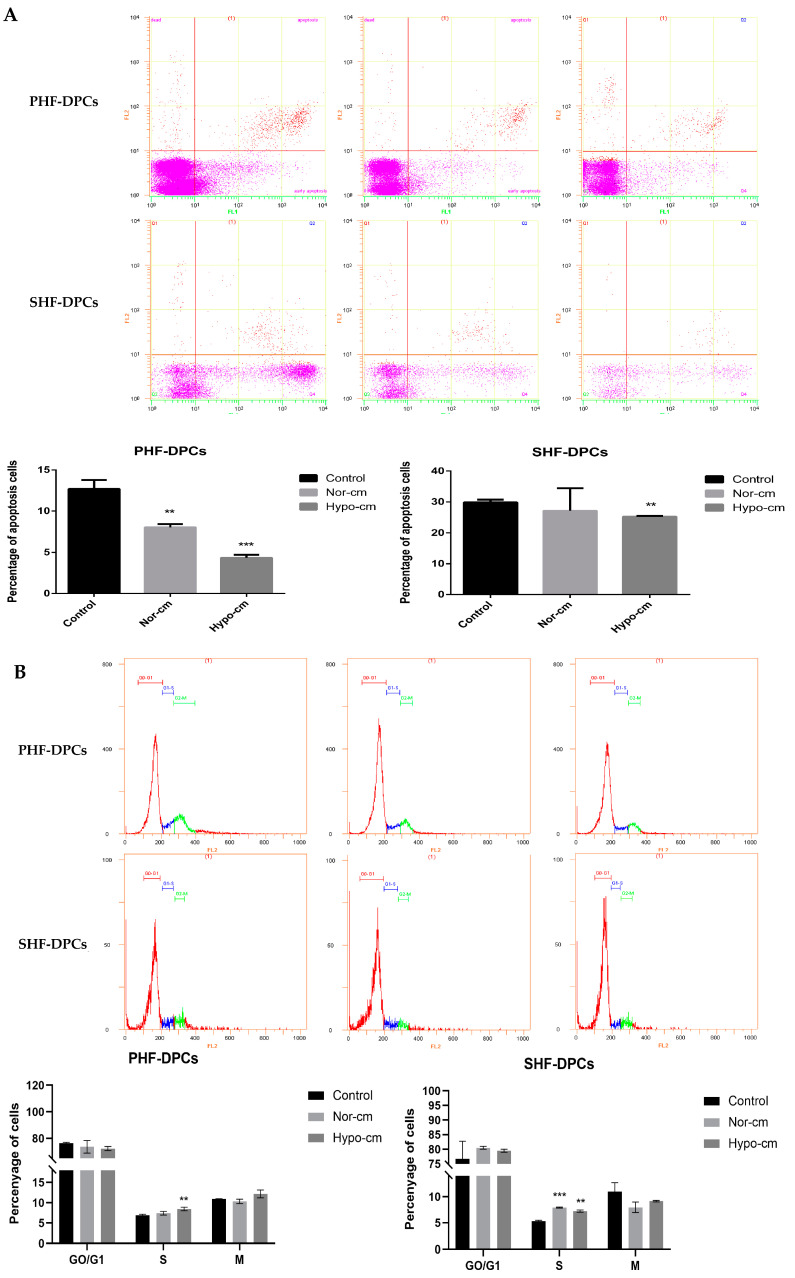
Conditioned medium for ADSCs enhances proliferation and induces follicle formation in DPCs. (**A**) Apoptosis was detected with flow cytometry. PHF-DPCs and SHF-DPCs were cultured for 48 h in Hypo-cm and Nor-cm and stained with Annexin V-FITC/PI to detect apoptosis. PHF-DPCs and SHF-DPCs were cultured for 48 h in Hypo-cm and Nor-cm. Distribution of apoptotic cells. N = 3. (**B**) Cell cycle was detected with flow cytometry. PHF-DPCs and SHF-DPCs were cultured in Hypo-cm and Nor-cm for 48 h, and PI staining was performed to determine the histogram distribution of each phase of the cell cycle. Number of cells in G0/G1, S, and M phases of PHF-DPCs and SHF-DPCs cultured for 48 h in Hypo-cm and Nor-cm. N = 3. ** 0.01 < *p* < 0.05; *** *p* < 0.01 compared with ADSCs cultured under normal conditions.

**Figure 6:**
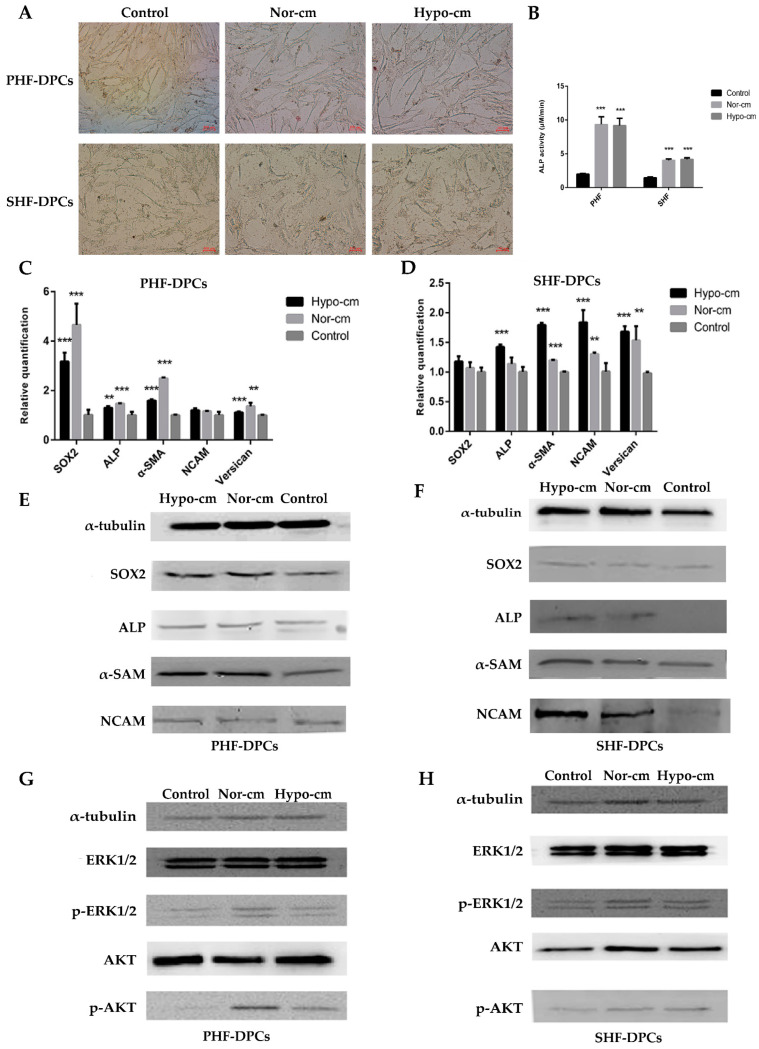
Conditioned medium for ADSCs enhances proliferation and induces follicle formation in DPCs. (**A**) *ALP* staining of PHF-DPCs and SHF-DPCs cultured for 48 h in Hypo-cm and Nor-cm (scale: 100 μm). N = 3. (**B**) *ALP* activity detection in PHF-DPCs and SHF-DPCs cultured in Hypo-cm and Nor-cm for 48 h. N = 3. (**C**,**D**) qRT-PCR results showing the transcription of specific marker genes *SOX2*, ALP, α-SMA, *NCAM*, and versican in PHF-DPCs and SHF-DPCs cultured for 48 h in Hypo-cm and Nor-cm. N = 3. (**E**,**F**) Western blot results showing the expressions of specific marker genes *SOX2*, ALP, α-SMA, and *NCAM* in PHF-DPCs and SHF-DPCs cultured for 48 h in Hypo-cm and Nor-cm. N = 3. (**G**,**H**) Western blot was used to detect activation of AKT and ERK signaling pathways in PHF-DPCs and SHF-DPCs cultured in Hypo-cm and Nor-cm. N = 3. ** 0.01 < *p* < 0.05; *** *p* < 0.01 compared with ADSCs cultured under normal conditions.

Both primary hair follicle–dermal papilla cells (PHF-DPCs) and secondary hair follicle–dermal papilla cells (SHF-DPCs) expressed the DPC-specific markers *CD133* and *α-SMA* after resuscitation (Figure 4A). DPCs added to Hypo-cm and Nor-cm media were assayed for the proliferation of Ki67 and EdU, and Hypo-cm expressed more Ki67 and had a higher rate of EdU-positive cells than the untreated DPCs (Figure 4B–E). These results suggest that the proliferation of DPCs can be improved using a conditioned medium culture, especially with Hypo-cm. In addition, results from a Western blot to detect the cell cycle protein *CDK2* showed that the use of a conditioned medium, especially Hypo-cm, increased the expression of *CDK2* in PHF-DPCs and SHF-DPCs (Figure 4F). The effect of the conditioned media on the hair papilla cell cycle was analyzed via flow cytometry. The proportion of S-phase cells increased after culturing PHF-DPCs and SHF-DPCs with Hypo-cm and Nor-cm compared with that of untreated DPCs; however, the differences in G0/G1 and M-phases were not significant (Figure 5A). The apoptotic distribution of DPCs was detected via flow cytometry, and the number of apoptotic cells was reduced after culturing both PHF-DPCs and SHF-DPCs using Hypo-cm and Nor-cm, compared to untreated DPCs. Also, Hypo-cm and Nor-cm had a greater and more significant effect on the apoptosis of PHF-DPCs (Figure 5B). ALP staining was performed on PHF-DPCs and SHF-DPCs cultured in a conditioned medium. The positive expression of ALP was higher in PHF-DPCs and SHF-DPCs after Hypo-cm and Nor-cm cultures than in untreated DPCs (Figure 6A). Furthermore, ALP activity was assayed in PHF-DPCs cultured in a conditioned medium versus SHF-DPCs. Compared with the control, the ALP activity rates of both PHF-DPCs and SHF-DPCs were significantly higher, regardless of whether Hypo-cm or Nor-cm was used (Figure 6B). The use of a conditioned medium increased the expressions of genes *ALP*, *SOX2*, *α-SMA*, *NCAM*, and versican (Figure 6C–F). This demonstrated that a conditioned medium, especially Hypo-cm, promoted the growth of DPCs and enhanced their ability to form hair follicles. Additionally, we investigated the signaling pathways that were altered in DPCs via the ADSC-conditioned medium. Western blot was performed to detect ERK1/2 and AKT phosphorylation levels. In PHF-DPCs and SHF-DPCs, the protein levels of total ERK1/2 and AKT were not altered following the use of Hypo-cm and Nor-cm, and the phosphorylation levels of phosphorylated ERK (p-ERK) and phosphorylated AKT (p-AKT) were higher than those of untreated DPCs (Figure 6G,H). This indicated that the conditioned medium activated the ERK1/2 and AKT signaling pathways in DPCs. Therefore, the increased proliferative capacity of DPCs is likely regulated via the ERK1/2 and AKT signaling pathways.

### 2.4. The ERK1/2 and HIF-1α Signaling Pathways Regulate Paracrine Levels in ADSCs in a Hypoxic Microenvironment

**Figure 7:**
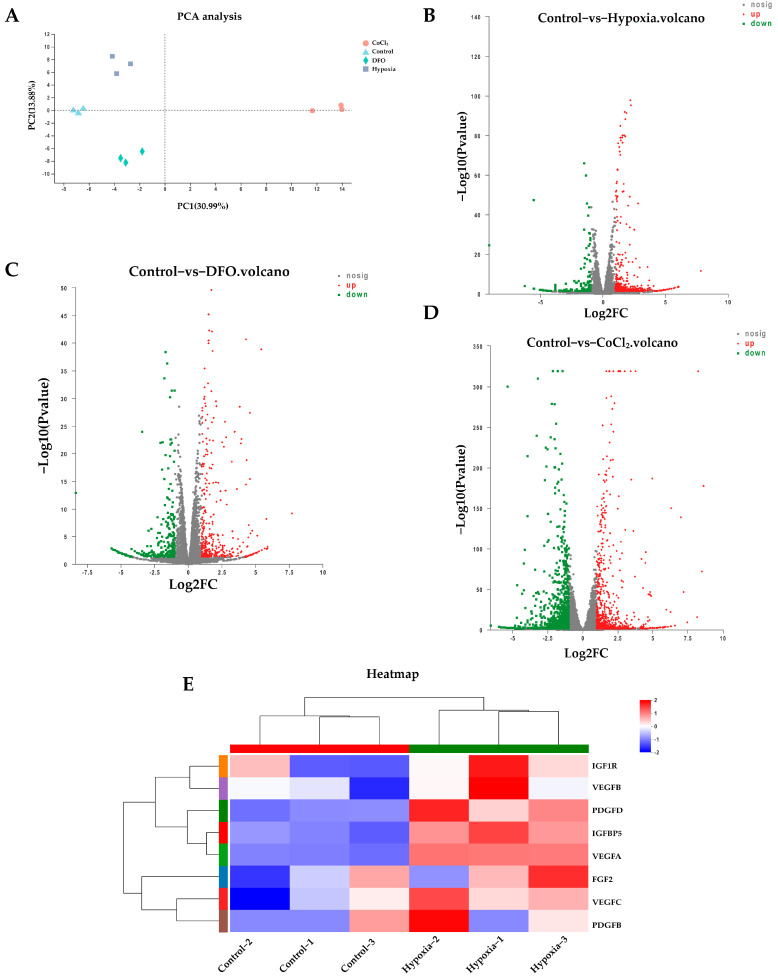
The ERK1/2 and *HIF-1α* signaling pathways regulate paracrine levels in ADSCs in a hypoxic microenvironment. (**A**) Principal component analysis (PCA) results showing the overall separation of ADSCs treated with hypoxia, DFO, and CoCl_2_ from untreated ADSCs. Each point represents RNA-Seq data from one organism. (**B**) “Hypoxia vs. control”, (**C**) “DFO vs. control”, and (**D**) “CoCl_2_ vs. control” volcano plot showing DEG analysis results. The x-axis is the log2 value (fold change), and the y-axis is the log10 value (*p*-value). Red dots show upregulated DEGs, and green dots show downregulated DEGs. (**E**) “Hypoxia vs. control” heatmap of the eight growth factor genes identified in the expression analysis table. Up-regulated (red) and down-regulated (blue) genes are shown as log2 (fold change) in hypoxia-treated and control groups. DEG, differentially expressed genes.

**Figure 8:**
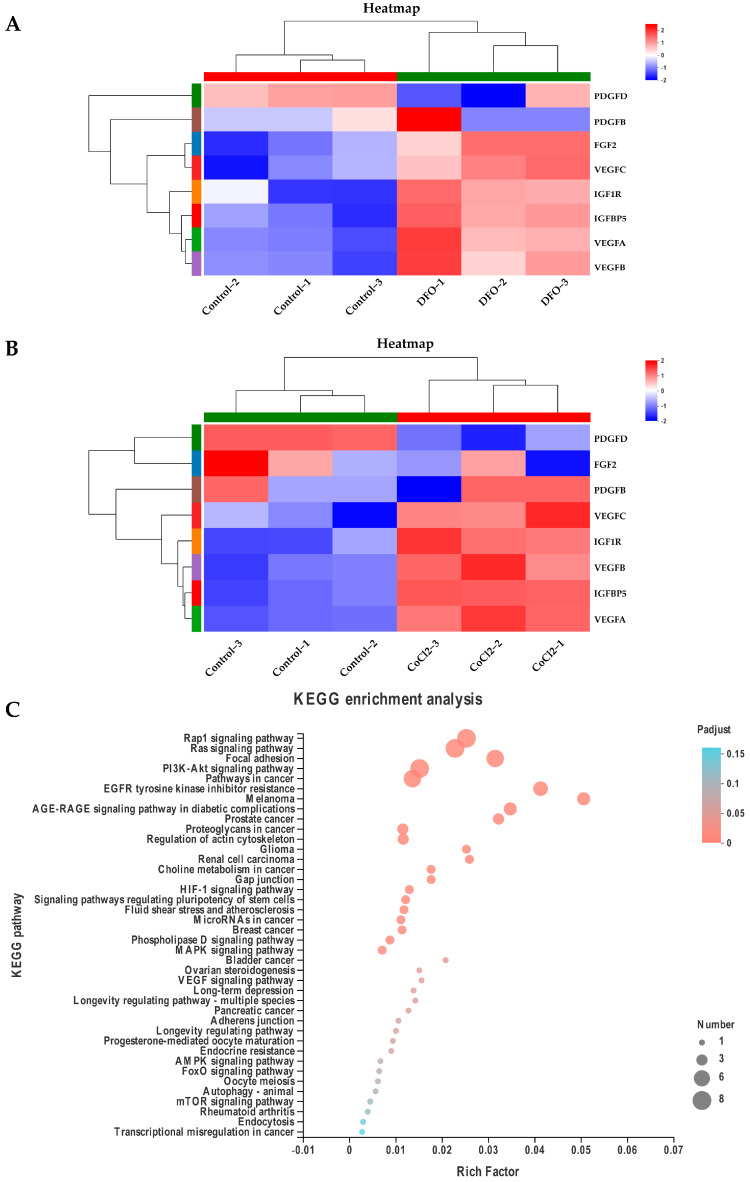
The ERK1/2 and *HIF-1α* signaling pathways regulate paracrine levels in ADSCs in a hypoxic microenvironment. (**A**) “DFO vs. control”, and (**B**) “CoCl_2_ vs. control” heatmap of the eight growth factor genes identified in the expression analysis table. Up-regulated (red) and down-regulated (blue) genes are shown as log2 (fold change) in hypoxia-treated and control groups. (**C**) KEGG enrichment bubble plots for the nine growth factor genes identified in the expression profile analysis table. KEGG, Kyoto Encyclopedia of Genes and Genomes.

**Figure 9:**
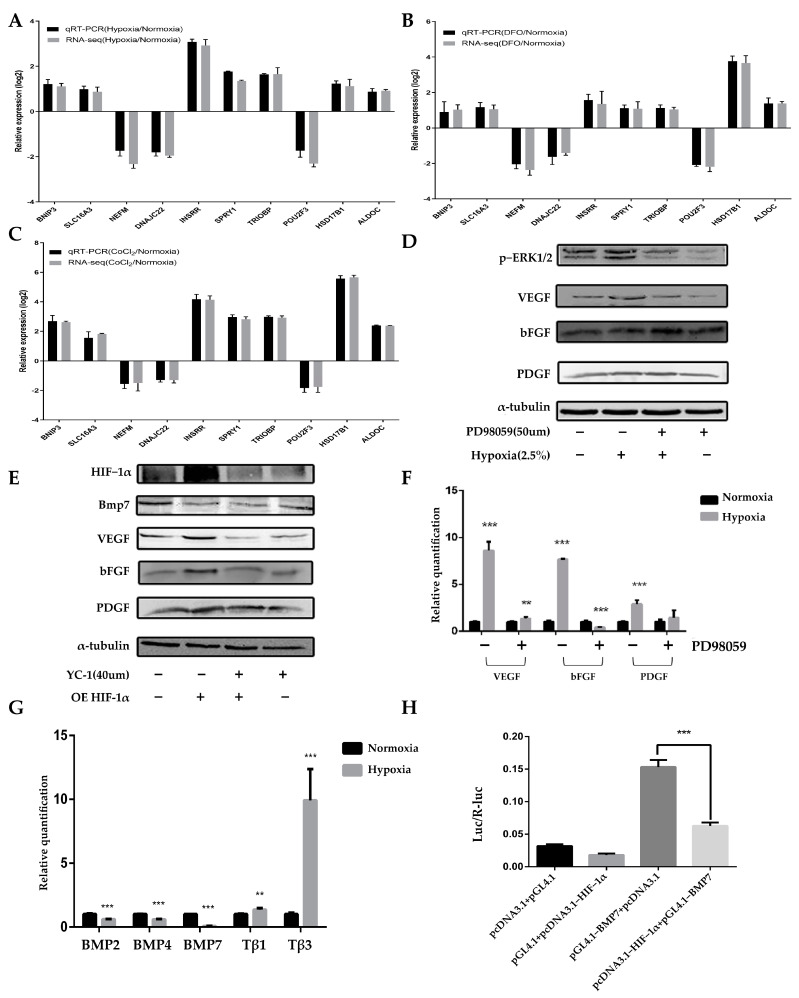
The ERK1/2 and *HIF-1α* signaling pathways regulate paracrine levels in ADSCs in a hypoxic microenvironment. RNA-seq results of the 10 DEGs of (**A**) “Hypoxia vs. control,” (**B**) “DFO vs. control,” and (**C**) “CoCl_2_ vs. control” validated with qRT-PCR. GAPDH was used as an internal control, and each value represents the mean of three independent biological replicates. N = 3. (**D**) Results of protein immunoblotting to detect the regulation of the ERK1/2 pathway via *VEGF*, *bFGF*, and *PDGF* at the protein level. N = 3. (**E**) Results of protein immunoblotting to detect the regulation of *Bmp7*, *VEGF*, *bFGF*, and *PDGF* expressions via overexpression of *HIF-1α* and addition of YC-1. N = 3. (**F**) Results of qRT-PCR to detect the regulation of ERK1/2 pathway depending on the expressions of *VEGF*, *bFGF*, and *PDGF* at the transcriptional level. N = 3. (**G**) Results of qRT-PCR to detect the effects of hypoxia incubation on the BMP gene family in ADSCs. N = 3. (**H**) Results of dual luciferase reporter assay to detect the effect of transcription factor *HIF-1α* on *BMP7* promoter activity. Differences between groups were analyzed with an unpaired *t* test. N = 3. ** 0.01 < *p* < 0.05; *** *p* < 0.01 compared with ADSCs cultured under normal conditions.

We investigated the effect of a hypoxic microenvironment on the transcriptome of ADSCs and determined the mechanism by which they promote the proliferation of DPCs. ADSCs treated with physical hypoxia, DFO, or CoCl_2_ for 48 h were analyzed using RNA-seq. Physical hypoxia, DFO, and CoCl_2_ samples were clearly separated from the control samples (Figure 7A). A total of 582 (hypoxia), 641 (DFO), and 1934 (CoCl_2_) genes were identified as differentially expressed genes (DEGs) (Figure 7B–D). The expressions of *VEGFA*, *VEGFB*, *VEGFC*, *PDGFB*, *PDGFD*, *FGF2 (bFGF)*, *IGFBP5*, and *IGF1R* increased in the hypoxia-treated group as per the transcriptome expression analysis table (Supplementary Table S3), *PDGFD* expression decreased in the DFO and CoCl_2_ groups, and *FGF2* (*bFGF*) expression decreased in the CoCl_2_ group (Figure 7E and Figure 8A,B). These genes were mainly enriched in the Rap1, Ras, MAPK, PI3K-Akt, and HIF-1 signaling pathways (Figure 8C). The quantitative reverse-transcription PCR (qRT-PCR) analysis was performed using specific primers. Ten DEGs were selected from three groups of ADSCs (hypoxia vs. control, DFO vs. control, and CoCl_2_ vs. control) for the qRT-PCR assay to verify the DEGs with FPKM (fragments per kilobase of transcript per million fragments mapped) values. Then, the qRT-PCR results were compared with FPKM values generated using RNA-seq. The qRT-PCR data were consistent with the RNA-seq data (Figure 9A–C). The hypoxic culture upregulated the expression of p-ERK1/2 in ADSCs compared to that in normoxic cultured ADSCs, and this upregulation was influenced by the ERK inhibitor PD98059 (Figure 9D). All three growth factors showed increased expression after hypoxic culturing, and the addition of PD98059 inhibited hypoxia-induced increases in *VEGF*, *bFGF*, and *PDGF* expression (Figure 9D,F). Western blot was also performed to detect *VEGF*, *bFGF*, and *PDGF* in the four groups of ADSCs overexpressing *HIF-1α* and the inhibitor YC-1 (Figure 9E). The overexpression of *HIF-1α* increased the expressions of *VEGF*, *bFGF*, and *PDGF* in ADSCs, with *VEGF* and *bFGF* increasing more significantly. In contrast, the addition of the *HIF-1α* inhibitor YC-1 inhibited this increase, and the expressions of *VEGF*, *bFGF*, and *PDGF* decreased significantly, indicating that their expression in ADSCs is regulated via *HIF-1α*.

Following the hypoxic culturing of ADSCs, the expression of proteins of the bone morphogenetic proteins (BMPs) family decreased, including BMP2, BMP4, and *BMP7*, with *BMP7* being the most significantly reduced (Figure 9G). Western blot results indicated a decrease in the *BMP7* expression following the overexpression of *HIF-1α* compared to that in the untreated control ADSCs. In contrast, *BMP7* expression was restored with the addition of the *HIF-1α* inhibitor YC-1, thereby suggesting that *HIF-1α* suppresses *BMP7* expression (Figure 9E). The results from the dual luciferase reporter gene assay showed a 2.37-fold reduction in promoter activity in the pGL4.1-*BMP7* and PcDNA3.1-*HIF-1α* groups compared with the transfected pGL4.1-*BMP7* and PcDNA3.1 groups (Figure 9F). This indicated that the transcription factor *HIF-1α* had a significant inhibitory effect on *BMP7* promoter activity.

## 3. Discussion

Oxygen levels in the bone marrow, brain, and adipose tissue are extremely low, at only 0.5–0.8%. Therefore, oxygen concentrations significantly affect the differentiation and survival of tissue cells. A hypoxic microenvironment can provide signals to maintain stem cell properties [31]. Previous studies suggest that the hypoxic microenvironment enhances angiogenesis mainly by improving the paracrine function of ADSCs and upregulating the expression of certain growth factors [8,32,33]. In this study, a hypoxic microenvironment increased the proliferative activity of ADSCs by promoting the expression of proliferative and anti-apoptotic genes and improving the migratory, angiogenic, and paracrine capacities of ADSCs. The use of conditioned media, especially Hypo-cm, promoted the growth of DPCs and enhanced their ability to form hair follicles. ERK1/2 is an important member of the mitogen-activated protein (MAPK) family with multiple functions, including the promotion of gene expression, proliferation, differentiation, and angiogenesis [34,35]. ERK1/2 is closely related to hair follicle growth and is a representative pathway for hair follicle growth stimulation. The activation of the ERK1/2 signaling pathway induces the proliferation of DPCs and inhibits apoptosis [36,37]. In addition, the ERK1/2 signaling pathway mediates *VEGF*-induced endothelial cell proliferation [38,39] and influences *VEGF*/*VEGF*R-2-mediated trichome growth [40]. The PI3K/AKT signaling pathway can induce cell proliferation and differentiation and inhibit apoptosis [41]. Our results showed that both p-ERK1/2 and p-AKT were significantly upregulated in DPCs cultured in a conditioned medium and that the ADSC-conditioned medium may promote hair follicle growth by activating the ERK1/2 and AKT signaling pathways in DPCs.

The results from Gene Ontology (GO) and Kyoto Encyclopedia of Genes and Genomes (KEGG) analyses showed that DEGs were significantly enriched in the chemokine signaling pathway and that ADSCs may respond to a hypoxic environment by increasing intercellular signaling via a chemokine-mediated signaling pathway in a hypoxic microenvironment [42,43,44]. The paracrine function of ADSCs strongly influences this process; still, the exact mechanisms involved in regulating the expression of the relevant factors are not clear. The results of the transcriptome analysis of ADSCs under a hypoxic microenvironment showed that this microenvironment increases the expression levels of growth factors such as *VEGFA*, *VEGFB*, *VEGFC*, *PDGFB, PDGFD*, *FGF2*, and *IGFBP5*. Only ERK is involved in the regulation of *VEGF* in glioblastoma multiforme under hypoxia [45]. Meldrum et al. also found that the ERK1/2 signaling pathway induces the secretion of large amounts of growth factors in MSCs [46,47,48,49,50]. ERK1/2 inhibitors decreased the expression of growth factors elevated by the hypoxic microenvironment; this suggests that the ERK1/2 signaling pathway is first activated via the hypoxic culture, which, in turn, induces the expressions of *VEGF*, *bFGF*, and *PDGF*. The hypoxic microenvironment may enhance the paracrine function of ADSCs by activating the ERK1/2 signaling pathway and ultimately promoting trichome growth. In this present study, the expressions of *VEGF*, *bFGF*, and *PDGF* similarly increased via ADSCs overexpressing *HIF-1α*; this increase was inhibited by the *HIF-1α* inhibitor YC-1, suggesting that hypoxia can induce *VEGF*, *bFGF*, and *PDGF* expressions via *HIF-1α*, thereby promoting hair follicle growth. In the hypoxic response, activated HIF-1 binds to the HRE (hypoxia-response element) site with the nucleotide sequence ACGTG to form the HIF-1-p300/CBP cyclic adenylate response element binding protein, which initiates gene transcription. Genes containing one or more HRE sites are called target genes, and the target genes are regulated via *HIF-1α* [51]. In this present study, the *HIF-1α* transcription factor had a significant inhibitory effect on *BMP7* promoter activity, which is preliminary proof of the reciprocal relationship between *HIF-1α* and *BMP7*, where *HIF-1α* binds to the HRE site in the promoter region of *BMP7* and directly regulates the expression of *BMP7*. Since members of the BMP family of proteins play a predominantly repressive role in hair follicle morphogenesis, the negative regulation of *BMP7* via *HIF-1α* further promotes hair follicle growth.

## 4. Materials and Methods

### 4.1. Cell Culture

Arbas cashmere goat adipose mesenchymal stem cells were isolated and identified in our laboratory [52], and the ADSCs used in this experiment were all 6th generation (passage). ADSCs were cultured in a DMEM/F12 culture medium containing 15% (*v*/*v*) fetal bovine serum at constant 37 °C and 5% CO_2_. After ADSCs were adhered, they were cultured under physical hypoxia (37 °C, 2.5% O_2_, 5% CO_2_) or hypoxia mimics (DFO and CoCl_2_) for 48 h. PHF-DPCs and SHF-DPCs from Arbas Cashmere goats were isolated in our laboratory [53], and all DPCs used in this experiment were 6th generation (passage). They were cultured in a DMEM/F12 culture medium containing 10% (*v*/*v*) fetal bovine serum at constant 37 °C and 5% CO_2_.

### 4.2. Cell Counting Kit-8 (CCK-8) Assay

The CCK-8 (Beyotime, Shanghai, China) was used to determine the viability of ADSCs from Arbas Cashmere Goats. ADSCs were inoculated into 96-well plates at a density of 1 × 10^3^ cells/well and placed in a hypoxic incubator with different concentrations of the hypoxia mimics DFO and CoCl_2_. Next, 24, 48, and 72 h after treatment, 10 μL CCK8 reagent was added to each well, and absorbance at 450 nm was measured after incubation for 2 h at 37 °C.

### 4.3. Western Blotting

Mammalian protein extraction was performed (CWBIO, Beijing, China). Protein concentrations were determined using the BCA protein assay kit (Thermo Fisher Scientific, Waltham, WA, USA). Proteins (20–30 μg) from cell lysates were separated via sodium dodecyl sulfate-polyacrylamide gel electrophoresis (SDS-PAGE) and electrophoretically transferred to nitrocellulose membranes (Pall). Membranes were blocked with 5% nonfat milk for 1 h at 20 °C and then incubated overnight at 4 °C with primary antibodies. After washing thrice the next day, the membranes were incubated with an HRP-conjugated secondary antibody (Abcam, Cambridge, UK, ab6802, 1:10,000) for 1 h at room temperature. Antigen-antibody complexes were visualized using Pierce ECL Western blotting substrate (Thermo Fisher Scientific). Membrane images were acquired using a Tanon 5200 microscope and analyzed using the ImageJ software. The primary antibodies used are listed in Supplementary Table S1.

### 4.4. qRT-PCR

Total RNA was extracted from ADSC using RNAiso (Takara, Dalian, China), and cDNA was synthesized using a PrimeScriptTM RT reagent kit with gDNA Eraser (Perfect Real Time) (Takara). PCR was performed using the CFX96 Real-Time PCR Detection System, which was employed for PCR and detection. Oleyl phosphate dehydrogenase (GAPDH) was used as a housekeeping gene, and the relative gene expression levels were calculated using the comparative CT method (ΔΔCT). The primers used in this study are listed in Table S2.

### 4.5. ELISA

The vascular endothelial cell growth factor ELISA kit (EIA06604Go, Xinqidi, Wuhan, China), basic fibroblast growth factor ELISA kit (EIA05081Go, Xinqidi, Wuhan, China), and goat platelet-derived growth factor ELISA kit (EIA06190Go, Xinqidi, Wuhan, China) were used for quantification of VEGF, bFGF, and PDGF, respectively, in conditioned media according to the manufacturer’s protocol. Briefly, ADSC supernatant was added to the reaction wells coated with antibodies. The plate was incubated at 37 °C for 90 min. Then, the plate was washed three times, and biotinylated antibody working solution was added. Next, the plate was incubated at 37 °C for 60 min and washed three times. Then, enzyme conjugate working solution was added, and the plate was incubated at 37 °C for 30 min and washed five times. TMB color development solution was added, the plate was incubated at 37 °C for no more than 30 min, and termination solution was added when there was a clear color gradient in the standard curve. Finally, the OD value (450 nm) was measured within 10 min of mixing.

### 4.6. Wound Healing Experiment

The effect of hypoxia on the migratory ability of ADSCs was investigated using a cell scratch assay as follows: ADSCs were inoculated at a density of 2 × 10^5^ cells/well in culture medium containing 1% (*v*/*v*) serum. Wounds were created 24 h later using a 1 mL pipette tip. Then, the cells were placed in a hypoxic or normoxic incubator for 24 h. After, the cells were incubated for 0, 6, and 12 h. Wounds were imaged under a microscope at 0, 6, 12, and 24 h. The relative migration rates were calculated based on wound closure and normalized to that of the control group.

### 4.7. Tube Formation Analysis

To observe the effect of hypoxia (physical hypoxia) on the angiogenic ability of ADSCs, they were cultured on Matrigel matrix gel (BD356234) using EGM-2 culture medium (Lonza, Walkersville, MD, USA), resuspended in EGM-2 culture medium, and inoculated into Matrigel matrix gel-treated 24-well plates containing 8 × 10^4^ cells/well. The cells were placed in a hypoxic or normoxic incubator and imaged under a microscope after 3 h.

### 4.8. Immunofluorescence Assay

ADSCs were seeded into 24-well plates and treated with 0.1% (*w*/*v*) gelatin (1 × 10^4^ cells/well) and incubated for 48 h. The cells were fixed with 4% paraformaldehyde at room temperature for 15 min, permeabilized with 0.5% (*v*/*v*) TritonX-100 at room temperature for 10 min, and sealed with 1% bovine serum albumin for 30 min. The primary antibody was incubated overnight at 4 °C. The secondary antibody conjugated with Alexa Fluor^®^ 488 (Abcam, ab150073) was diluted at a 1:300 ratio and incubated for 1 h at room temperature. After staining with DAPI for 5 min, the slices were sealed with glycerol, and images were taken for staining with confocal laser microscopy. The antibodies used are listed in Table S2.

### 4.9. Flow Cytometry

PHF-DPCs were inoculated into 6-well plates at 8 × 10^4^ cell/well and SHF-DPCs at 1 × 10^5^ cell/well. DPCs were grown in Hypo-cm- and Nor-cm-conditioned media. Normal untreated cultures were used as controls. Cells were treated with a Cell Cycle and Apoptosis Assay Kit (7sea biotech, C001, Shanghai, China) and an Annexin V-FITC/PI Double-stained Apoptosis Assay Kit (7sea biotech, A005, China). After 48 h, DPCs cultured in conditioned medium were collected in 1.5 mL centrifuge tubes, and the cells were washed with pre-cooled PBS and fixed in 70% (*v*/*v*) anhydrous ethanol for 1 h. After washing again with PBS, 1 mL staining solution was prepared, 20 μL RNAase and 25 μL PI were added, and the cells were gently mixed, resuspended, and incubated for 30 min at 37 °C protected from light. Then, changes in the cell cycle and apoptosis were detected using flow cytometry. Data were further analyzed using FlowJo software (FlowJo LLC, Ashland, OR, USA).

### 4.10. Cell Proliferation Assay

PHF-DPCs and SHF-DPCs were inoculated into 4-well plates containing 2.5 × 10^4^ cells/well and cultured in Hypo-cm and Nor-cm conditioned media, respectively, using cells in untreated culture medium as the control. Cells were treated according to the instructions of the EdU cell proliferation assay kit (Reebok Apollo567). Briefly, 100 μL of 50 μM EdU medium were added to each well, and the plate was incubated for 2 h. The medium was discarded, 100 μL PBS was added, and the plate was shaken for 5 min. Then, 50 μL of 4% paraformaldehyde was added to fix at room temperature for 30 min; then, the paraformaldehyde was discarded. Additionally, 50 μL of 2 mg/mL glycine was added, and the plate was shaken for 5 min. The glycine was discarded, 100 μL PBS were added, and the plate was shaken for 5 min. Moreover, 100 μL of 1× Apollo staining solution was added to each well. Then, the plate was incubated for 30 min at room temperature protected from light. The staining solution was discarded, and 100 μL of 0.5% TritonX-100 was added. The plate was shaken 2–3 times for 10 min each time, and the permeate was discarded. Next, 100 μL 1× Hoechst33342 reaction solution was added, and the plate incubates for 30 min at room temperature and protected from light. Finally, the cells were washed 1–2 times with PBS, and the laser confocal microscope was used to take pictures after the staining was complete to observe cell proliferation.

### 4.11. ALP Staining and Activity Assay

PHF-DPCs were inoculated into 6-well plates at 1 × 10^5^ units/well and SHF-DPCs at 1.5 × 10^5^ units/well, using Hypo-cm or Nor-cm media. Cells in untreated culture medium were used as a control. An alkaline phosphatase staining kit (G1481, Solarbio, Beijing, China) and an alkaline phosphatase activity assay kit (21101ES60, Yeasen Biotechnology, Shanghai, China) were used to detect the staining and activity of the conditioned media for PHF-DPs and SHF-DPC ALP markers.

The DPCs and media were discarded, and the plate was washed with PBS 1–2 times. Then, 300 μL ALP fixative was added to each well for 3 min, followed by washing 1–2 times with PBS. Next, the configured ALP incubation solution was added dropwise, and the plate was incubated for 20 min avoiding light. The plate was washed 1–2 times with PBS, 300 μL nuclear solid red staining solution was added for 5 min, and the plate was washed 1–2 times with PBS. Then, photographs were taken under an inverted microscope to observe the staining.

Cells were lysed using 0.1% (*v*/*v*) TritonX-100 at 4 °C overnight. Blank control, standard, and sample wells were set up. The three reaction solutions were mixed gently and incubated at 37 °C for 10 min. Next, 100 μL of reaction termination solution was added to each well, and the absorbance at 405 nm was measured with a multifunctional microplate reader to assess ALP activity.

### 4.12. Cell Transfection and Plasmid Construction

Cell transfection was performed using Lipofectamine 2000 (Invitrogen, Carlsbad, CA, USA), according to the manufacturer’s protocol. The plasmids (3 μg) were incubated in Opti-MEM (Gibco, NY, USA) solution for 5 min; 9 µL Lipofectamine 2000 was mixed into the solution, which had been incubated for 20 min. The mixtures were added to the cells and incubated for 6 h at 37 °C. Then, the cells were cultured in 10% (*v*/*v*) fetal bovine serum for 48 h. The *HIF-1α*-CDS expression plasmid was generated by cloning the full-length ORF of the goat *HIF-1α* gene (XM_018053545.1) into the pCMV6-AC-IRES-GFP vector. Primers were synthesized via Huada Gene, and the primers used in this study are listed in Table S2.

### 4.13. Dual Luciferase Reporter Assay

The BMP7 promoter and HIF-1α sequences were ligated to the PGL4.10 and PcDNA3.1-3FLAG vectors to generate the corresponding BMP7 luciferase reporter vector pGL4.1-BMP7 and expression vector PcDNA3.1-HIF-1α, respectively. Following 48 h of transfection, the dual luciferase reporter gene was assayed using a Dual Luciferase Reporter Gene Assay Kit (Promega, Madison, WI, USA) to detect firefly and lake kidney luciferase activities.

A mixture of F. fluorescens fluorophore reporter vector, sea kidney fluorophore vector, and transfection reagent was prepared in a ratio of 0.1 μg:0.005 μg:0.25 μL, and XX per well was added. A mixture of transcription factor or control plasmid and transfection reagent in the ratio of PcDNA3.1-HIF-1 was prepared. After diluting the luciferase reporter vector, transcription factor plasmid, and transfection reagent, the cells were incubated for 5 min at room temperature, and the diluted luciferase reporter vector and transcription factor vector were mixed with transfection reagent and incubated for 20 min at room temperature. After 6 h of transfection, the cells were replaced with new cell culture medium. After 48 h of transfection, the medium was discarded, and the cells were washed once with 100 μL PBS to remove as much PBS as possible. A 5× passive lysis buffer (PLB) was diluted into 1× PLB with deionized water. Additionally, 100 μL of diluted 1× PLB lysis solution was added and incubated for 20 min at room temperature in a shaker for lysis. Then, 5 μL of lysis product was added, followed by the addition of 50 μL of pre-mixed LARII reagent; the firefly photon count was measured after 2 s. Next, 50 μL of pre-mixed Stop&Glo reagent was added, and the sea kidney photon count was measured after 2 s. This experiment was performed under light-proof conditions throughout.

### 4.14. Transcriptome Analysis

ADSCs were collected after hypoxic (2.5% O_2_), DFO (25 μM), CoCl_2_ (150 μM), and normoxic (5% O_2_) conditions for 48 h. Three replicates of each group were selected, and total RNA was extracted from the cell samples using TRIzol reagent (Invitrogen, CA, USA). Sequencing libraries were prepared according to the RNA sample preparation kit protocol (Illumina, San Diego, CA, USA). Double-end sequencing was performed using an Illumina Novaseq 6000 2100 platform (Agilent, Santa Clara, CA, USA). Raw data were filtered using fastp (version 0.19.5) to trim the splice sequences and obtain high-quality clean reads. A reference genome index was created using hisat2 software v. 2.1.0, and clean reads were mapped to the reference genome (version: ARS1). To compare the expression levels of a given transcript between different samples, we quantified the relative expression of all transcripts using RSEM (version v1.3.1). FPKM was chosen as the scoring index, and differential gene expression analysis was performed using the edgeR software. The fold change (FC) was set at 2.0, |log2FC| ≥ 1, and the significance level was set at *p* < 0.05. The Benjamini–Hochberg (B-H) multiple test correction method was used for separate statistical tests and acted as a separate correction for false positives (*p* < 0.05, considered significant enrichment).

### 4.15. Statistical Analysis

Experimental data were analyzed using SPSS software. All data were subjected to a normality test (Shapiro–Wilk test) and presented as the mean ± standard deviation. All data were normally distributed; thus, statistically significant differences between two groups were determined using a *t*-test. *p*-values < 0.05 were considered statistically significant.

## 5. Conclusions

A hypoxic microenvironment in ADSCs promotes the growth of DPCs primarily via the ERK1/2 signaling pathway and *HIF-1α*, which regulated the expression of growth factors *VEGF, bFGF*, and *PDGF*. These results provide a better understanding of the molecular regulatory mechanisms underlying the effects of ADSCs on DPCs. The exact functions and mechanisms by which ADSCs promote the ability of DPCs to induce hair follicle growth are of high clinical value. Future efforts will aim to understand whether ADSCs acting on DPCs in hypoxic microenvironments via growth factors represent a common, therapeutically tractable, pathway that mediates the proliferation of DPCs in both physiological and pathological settings.

## Data Availability

The datasets used and analyzed in this study are available from the corresponding author upon reasonable request.

## Supplementary Materials

The supporting information can be downloaded at: https://www.mdpi.com/article/10.3390/ijms241311198/s1.

## Abbreviations

ADSCs	adipose mesenchymal stem cells
DPCs	dermal papilla cells
Hypo-cm	hypoxia-conditioned medium
Nor-cm	normoxic-conditioned medium
*HIF-1α*	hypoxia-inducible factor-1α
ADSC-CM	adipose mesenchymal stem cells -conditioned medium
DFO	desferrioxamine
CoCl_2_	cobalt chloride
ALP	alkaline phosphatase
BMPs	bone morphogenetic proteins
PHF-DPCs	primary hair follicle–dermal papilla cells
SHF-DPCs	secondary hair follicle–dermal papilla cells
CCK-8	Cell Counting Kit-8
QRT-PCR	quantitative reverse transcription polymerase chain reaction
EGM-2	endothelial cell growth medium-2
BCA	bicinchoninic acid
DAPI	4′,6-diamidino-2-phenylindole
FPKM	fragments per kilobase of transcript per million fragments mapped
MAPK	mitogen-activated protein kinase
DEGs	differently expressed genes
GBM	glioblastoma multiforme
HRE	hypoxia response element
