# Supplementary material for: Hypoxia Treatment of Adipose Mesenchymal Stem Cells Promotes the Growth of Dermal Papilla Cells via HIF-1α and ERK1/2 Signaling Pathways
Source: Int J Mol Sci. 2023 Jul 7;24(13):11198. doi: 10.3390/ijms241311198 (PMC10342612; doi:10.3390/ijms241311198)
Supplement: Supplementary file 1 [file ijms-24-11198-s001.zip › Table S1.pdf]

Supplementary Table S1. The information of antibodies used in this study. Related to Figure 1-9

Antibodies	Source	Identifier	Host	Proportion
Anti -AKT	Cell Signaling Technologies	#9272	Rabbit	1:1000
Anti -P-AKT	Cell Signaling Technologies	#9271	Rabbit	1:1000
Anti -ERK	Cell Signaling Technologies	#4695	Rabbit	1:1000
Anti -P-ERK	Cell Signaling Technologies	#4370	Rabbit	1:1000
Anti- HIF-1α	Cell Signaling Technologies	#3716	rabbit	1:1000
Anti -Nanog	Abcam	ab21624	rabbit	1:500
Anti -OCT4	Abcam	ab137427	rabbit	1:500
Anti -SOX2	Abcam	ab97959	rabbit	1:500
Anti -TERT	Abcam,	ab32020	rabbit	1:500
Anti -PCNA	Abcam	ab92552	rabbit	1:500
Anti -p53	Abcam	ab32389	Rabbit	1:500
Anti -Bax	Abcam	ab32503	Rabbit	1:500
Anti -Bcl2	Abcam	ab182858	Rabbit	1:500
Anti -CD44	Abcam	ab189524	Rabbit	1:1000
Anti -CD90	Abcam	ab133350	Rabbit	1:1000
Anti -CD34	Abcam	ab81289	Rabbit	1:1000
Anti -CD45	Abcam	ab40763	Rabbit	1:1000
Anti -ALP	Abcam	ab229126	Rabbit	1:1000
Anti -NCAM	Abcam	ab133345	Rabbit	1:1000
Anti-α-SMA	Abcam	ab124964	Rabbit	1:1000
Anti -CD133	Abcam	ab19898	Rabbit	1:1000
Anti -KI67	Abcam	ab15580	Rabbit	1:1000
Anti -CDK2	Abcam	ab32147	Rabbit	1:1000
Anti -BMP7	Abcam	ab129156	Rabbit	1:1000
Anti -VEGF	Abcam	ab46154	Rabbit	1:1000
Anti -PDGF	Abcam	ab23914	Rabbit	1:1000
Anti -bFGF	Abcam	ab208687	Rabbit	1:1000
Anti-α-tubulin	Abcam	ab52866	Rabbit	1:1000
