# Supplementary material for: Hypoxia Treatment of Adipose Mesenchymal Stem Cells Promotes the Growth of Dermal Papilla Cells via HIF-1α and ERK1/2 Signaling Pathways
Source: Int J Mol Sci. 2023 Jul 7;24(13):11198. doi: 10.3390/ijms241311198 (PMC10342612; doi:10.3390/ijms241311198)
Supplement: Supplementary file 1 [file ijms-24-11198-s001.zip › Table S2.pdf]

Supplementary Table S2. The information on the sequences of the primers used in this study. Related to Figure 1-9

Gene Name		Sequence	Product Length (bp)
NANOG	F	5'-GTCTCTCCTCTTCCTTCCTCCA-3'	116
	R	5'-TCTTCCTTCTCTGTGCTCTCCTC-3'	
OCT4	F	5'-GCCAAGCTCCTAAAGCAGAAGA-3'	122
	R	5'-AAAGCCTCAAAACGGCAGATAG-3'	
SOX2	F	5'-CATGATGGAGACGGAACTGG-3'	44
	R	5'-CGGGCTGTTCTTCTGGTTG-3'	
TERT	F	5'-TGAGCTTTGCCCACTGCAT-3'	112
	R	5'-TGCTGCCTGACCTCTGCTT-3'	
PCNA	F	5'-TTCGTAATCGTGTTTTGCCTCT-3'	158
	R	5'-CGCCTCCAGCACTTTCTTC-3'	
p53	F	5'-TGCACCACCATCCACTACAAC-3'	143
	R	5'-GCACAAACACGCACCTCAA-3'	
BAX	F	5'-CCTCTTCCCTCCTTTCTCCTCT-3'	99
	R	5'-AACATTTCAGCCGCCACTC-3'	
Bcl-2	F	5'-TGTGGATGACCGAGTACCTGAA-3'	120
	R	5'-AGACAGCCAGGAGAAATCAAACA-3'	
ALP	F	5'-GCCCTCTCCAAGACATACAACA-3'	80
	R	5'-TTCACCCCGCACAAGTAGG-3'	
SOX2	F	5'-CATGATGGAGACGGAACTGG-3'	44
	R	5'-CGGGCTGTTCTTCTGGTTG-3'	
α-SMA	F	5'-AGAGCATCCAACCCTTCTCAC-3'	120
	R	5'-ACCGCCTGAATAGCCACATAC-3'	
NCAM	F	5'-TCCTCCGCATCCTACACCTT-3'	213
	R	5'-CGCCAAACCTTCCTCTTCC-3'	
Versican	F	5'-AGGTGGTTTACTTGGGGTGAGA-3'	129
	R	5'-TTGAGGCAAGGGTTTGTTTTG-3'	
GAPDH	F	5'-TTCCACGGCACAGTCAAGG-3'	184

	R	5’-CTCAGCACCAGCATCACCC-3’	
VEGF	F	5’- CATTGAGACCCTGGTGGACAT -3’	184
	R	5’- CTGGCTTTGGTGAGGTTTGAT -3’	
bFGF	F	5’- AGAAGAGCGACCCTCACATCA -3’	226
	R	5’- CGTTTCAGTGCCACATACCAA -3’	
PDGF	F	5’- GTGACCACTCCATCCGTTCCTT -3’	248
	R	5’- GAAGTTAGCATTGGTGCGGTCT -3’	
BMP2	F	5’- ATCCATTTCCTCATTCTCCCAA-3’	246
	R	5’- CAACCGCCTGAACATCTCCTCT-3’	
BMP4	F	5’- ATCACACGACTACTGGACACA-3’	203
	R	5’- CCACTCCCTTGAGGTAACGAT-3’	
BMP7	F	5’- AGGAGGGCTGGCTCGTTTT-3’	120
	R	5’- TGGGGTTGATGCTCTGTCC-3’	
Tβ1	F	5’- TGACCCACAGAGAGGAAATAGA-3’	84
	R	5’- TGTCCACTTGAAGCGTGTTATC-3’	
Tβ3	F	5’- TTTACAACAGCACCCGAGAGC -3’	229
	R	5’- CGGAACAGGTTGGTTTCATTT -3’	
HIF-1α	F	5’-ACCGATTCACCATGGAGGGC-3’	2649
	R	5’-GCTCAGTTAACTTGATCCAAAGC-3’	
SLC16A3	F	5’- ACTGGGGAAGTGCCGC-3’	115
	R	5’- CTGATTCCGTGTCCCTGCTG-3’	
NEFM	F	5’- GATCGCCGCATACAGGAAACT-3’	159
	R	5’- TGGACCTTTAACTTGGGAGCC-3’	
DNAJC22	F	5’- TGGTCGGAGATCATGGCATC-3’	118
	R	5’- GAGAGACCAAAAACCTGGAGAG-3’	
INSRR	F	5’- CAGCCAACCCTGGTCATCAT-3’	255
	R	5’- AGTCATCCCGAAGTCCCCAA-3’	
ALDOC	F	5’- TCAAGCGGGCTGAGGTGAAT-3’	298
	R	5’- GCTCCCTATCCTCCCATCCA-3’	

HSD17B1	F	5’- AGGTGGTCGAGGTCTTCCT-3’	212
	R	5’- TCTCAGTTTACCGGCTTCGG-3’	
TRIOBP	F	5’- TCAGTGGCCGAAGACCAAAG-3’	134
	R	5’- CCTTTGATTGGAACTCCCGC-3’	
SPRY1	F	5’- GTTGATGCGGCGAGTGC-3’	100
	R	5’- ATGCATCTGAAATCCTAGGCCG-3’	
BNIP3	F	5’- GCTGCTTATCTGCTGTGCG-3’	219
	R	5’- TAACTCTACCCAGGAGCCGTG-3’	
POU2F3	F	5’- AGCCAGGTGGAGACAGATTA-3’	100
	R	5’- CATCATGGCAGGTCCTTGACT-3’	
